# Prevalence of tobacco use among cancer patients in Iran: a systematic review and meta-analysis

**DOI:** 10.1186/s12889-024-18594-8

**Published:** 2024-04-18

**Authors:** Mohammad Moein Vakilzadeh, Reza Khayami, Danyal Daneshdoust, Reza Moshfeghinia, Farzad Sharifnezhad, Zahra Taghiabadi, Hanieh Keikhay Moghadam, Mohammad Ali Karimi, Atousa Ghorbani, Pegah Bahrami Taqanaki, Nima Boojar, Adele Azarshab, Soodabeh Shahidsales, Reihaneh Alsadat Mahmoudian

**Affiliations:** 1 Cancer Research Center, Mashhad University of Medical Sciences, Mashhad, Iran; 2 Faculty of Medicine, Babol University of Medical Sciences, Babol, Iran; 3 Student Research Committee, Shiraz University of Medical Sciences, Shiraz, Iran; 4 Department of Internal Medicine, Mashhad University of Medical Science, Mashhad, Iran; 5 Department of Microbiology and Virology of Medicine, Mashhad University of Medical Science, Mashhad, Iran; 6 Student Research Committee, Varastegan Institute for Medical Science, Mashhad, Iran; 7 Student Research Committee, Mashhad University of Medical Sciences, Mashhad, Iran; 8 Department of Biology, North Tehran Branch, Islamic Azad University, Tehran, Iran; 9 Faculty of Medicine, Mashhad University of Medical Sciences, Mashhad, Iran; 10 Metabolic Syndrome Research Center, Mashhad University of Medical Sciences, Mashhad, Iran

**Keywords:** Cancer, Tobacco products, Smokeless tobacco, Public health, Middle East

## Abstract

**Background:**

The prevalence of tobacco use among various cancer types in Iran remains a significant concern, necessitating a comprehensive analysis to understand the extent and patterns of consumption. This study aimed to systematically review and analyze existing literature to delineate the prevalence of tobacco use across different cancer types in Iran, thereby providing a robust basis for future interventions and policy formulations.

**Methods:**

Adhering to the PRISMA guidelines, we conducted a systematic review and meta-analysis of literature available in PubMed and Scopus databases. The initial search identified 351 records, out of which 44 studies were selected based on their relevance and design. These studies spanned various time frames, starting from the 2001s up until 2022, and encompassed diverse geographical locations and cancer types in Iran. To avoid bias and potential data overlap, we opted to incorporate a single comprehensive study from the Golestan Cohort, encompassing all data, while excluding 10 other studies. Our final analysis incorporated data from 34 studies, which accounted for 15,425 patients and 5,890 reported smokers. Statistical analyses were performed to calculate the overall proportion of tobacco consumption and to conduct subgroup analyses based on different variables such as cancer types, gender, geographical locations, and types of tobacco used.

**Results:**

The analysis revealed a substantial prevalence of tobacco use among cancer patients in Iran, with an overall consumption rate of 43%. This rate varied significantly, ranging from 10 to 88% across individual studies. Subgroup analyses further highlighted disparities in tobacco consumption rates across different demographics, geographic areas, and cancer types. Notably, the ‘ever’ smokers category exhibited the highest prevalence of tobacco use. The study also identified a worrying trend of high cigarette smoking rates, along with variable consumption patterns of other forms of tobacco, including waterpipe, ‘Naas’, and ‘Pipe’.

**Conclusions:**

This systematic review and meta-analysis underscores a significant association between tobacco consumption and various cancer types in Iran, with a prevalence rate among cancer patients being three times higher than the average Iranian population. The findings indicate substantial heterogeneity in tobacco use patterns, emphasizing the need for targeted interventions to address this pressing health issue. The study serves as a critical resource for shaping future policies and strategies aimed at curbing tobacco use and mitigating its adverse effects on cancer prevalence in Iran.

## Introduction

Cancer is the second group of chronic non-communicable disease and ranks as the third most prevalent source of mortality in Iran [1]. Five prevalent types of cancer that affect Iranian men include stomach, prostate, bladder, colorectal, and esophagus. On the other hand, for Iranian women, the most common types of cancer are breast, colorectal, stomach, esophagus, and thyroid [2]. It is noteworthy that there is a discernible upward trajectory in the frequency and fatality rate of the majority of cancers in Iran [3].

Smoking in individuals results in a significant elevation of cancer risk, and it has been confirmed that tobacco smoke contains over 60 well-established carcinogens [4]. Tobacco smoking is a causative agent for multiple types of cancers that affect various parts of the human anatomy, including but not limited to the oral cavity, lung, pharynx, esophagus, kidney, colon, stomach, pancreas, bladder, rectum, liver, larynx, cervix, ureter, and bone marrow [5]. Despite being diagnosed with cancer, the continuation of tobacco use has been found to significantly elevate the likelihood of experiencing treatment-related toxicities, recurrence of cancer, morbidity, and mortality [6].

Smoking is known to elevate the concentration of carbon monoxide in the blood, leading to a reduction in the pulmonary air capacity. Consequently, smokers experience premature exhaustion in contrast to their non-smoking counterparts [7]. According to estimates, smoking is responsible for the untimely deaths of over fifty percent of individuals who engage in it over a prolonged period of time [8]. The mortality rate among individuals who smoke cigarettes across all age cohorts is observed to be 2–3 times greater compared to those who do not partake in smoking [9].

Diagnosing and treating cancer can lead to rapid cessation of smoking so that those with smoking-related cancers are more likely to quit [10]. The evidence pertaining to smoking and its correlation with cancer holds significant ramifications for public health with regards to the prevention of cancer, a condition that could potentially be rectified through the modification of individual behavioral patterns [11, 12]. The alteration in individuals' manner of living and environment can potentially impact the epidemiological trends pertaining to distinct forms of cancer [13]. According to the fact that smoking constitutes the most significant avoidable factor leading to cancer in multiple nations, the considerable shifts in smoking incidence in Iran necessitate a reevaluation of the present condition of malignancies related to tobacco [14]. Therefore, we conducted a comprehensive examination of the existing literature through a systematic review and meta-analysis of the prevalence of tobacco exposure in population who suffer from cancer in Iran.

## Methods

We have meticulously adhered to the Guidelines for Accurate and Transparent Health Estimates Reporting, as well as to the rigorous standards outlined in the Preferred Reporting Items for Systematic Reviews and Meta-Analyses [15, 16].

### Search strategy

We performed a comprehensive search to find studies published electronically between 21/03/2001 and 28/01/2023, based on articles available in Pubmed and Scopus databases. keywords and the Medical Subject Heading (MeSH) terms included Cigarette OR “Tobacco” OR “Pipe” OR “Cigar” OR “Hookah” AND Iran OR “Iranian” AND Cancer OR “Tumor” OR “Neoplasm” OR “Neoplasia” OR “Malignancy” OR “Malignancies” OR “Malignant” OR “Benign” Moreover, various types of observational studies, including cohort, cross-sectional, and case–control studies were considered.

### Criteria for selection and evaluation of quality of the papers

The search process was executed by two authors, so that they could issue a definitive verdict after careful discussion. The relevant title and abstract of the papers have been carefully examined for having acceptable quality of papers using the STROBE checklist (Strengthening the Reporting of Observational Studies in Epidemiology). Data were extracted on description of cancer type, sample size, study province, study period and data collection method. The aim of this study was to determine the correlation between smoking and the development of cancer. Consequently, the inclusion criteria involved providing an accurate estimation of the frequency of tobacco use among Iranian cancer patients.

### Data extraction

A form comprising of several sections was devised, and fundamental information necessary for the research was gathered. The required information involved the subject, title, journal’s name, and author, methodology and study design, prevalence of cigarette smoking among cancer patients, cancer type, study province, number of smokers by sex, and type of tobacco use.

### Study risk of bias assessment

In order to evaluate the trustworthiness and applicability of the studies included in our review, we undertook a comprehensive assessment of the risk of bias. This process was independently carried out by two reviewers using the Newcastle–Ottawa Scale (NOS). This tool is specifically designed to scrutinize the quality of non-randomized studies, especially observational ones. It takes into consideration three broad aspects: the method of selection for study groups, the degree of comparability between these groups, and the determination of either the exposure or outcome of interest. The NOS allowed us to score each study based on these criteria, leading to classifications of low, high, or unclear risk of bias. In case of any disagreements between the two reviewers, the issue was discussed thoroughly until a mutual agreement was reached.

### Data synthesis

Eligibility for each synthesis was determined based on how well the studies aligned with our pre-established inclusion criteria. We categorized studies according to their intervention characteristics, then compared these with the groups we had planned for each synthesis. All included studies in our analysis provided data on both smokers and non-smokers. Subgroup analyses were restricted to studies that specifically reported data for the relevant subgroups and those with missing data were not included. Forest plots, funnel plots, and risk of bias plots were utilized for the graphical representation and tabulation of individual studies and their syntheses.

Due to the high degree of heterogeneity among studies, we used a random effects model for meta-analysis with a random intercept logistic regression method [17]. Furthermore, mixed effect logistic regression models were used to analyze the effect of cigarette and cancer type alone and combined. We used the I^2^ statistic and the Chi^2^ test with a *p*-value less than 0.1 to identify statistical heterogeneity. To explore possible causes of heterogeneity, we carried out subgroup analyses based on factors such as cancer type, gender, geographical location and tobacco consumption habits. Furthermore, we performed sensitivity analyses to assess the stability of our synthesized results, whereby we sequentially removed each study and observed the resulting impact on the overall effect size.

### Assessment of reporting bias

In order to gauge the potential bias arising from absent results, we employed Egger’s regression test and conducted a visual inspection of funnel plots for any signs of asymmetry. In theory, studies with significant effects should symmetrically surround the aggregated effect size. If there is any departure from this expected symmetry, it indicates a possible presence of publication bias.

## Results

### Study selection

**Fig. 1:**
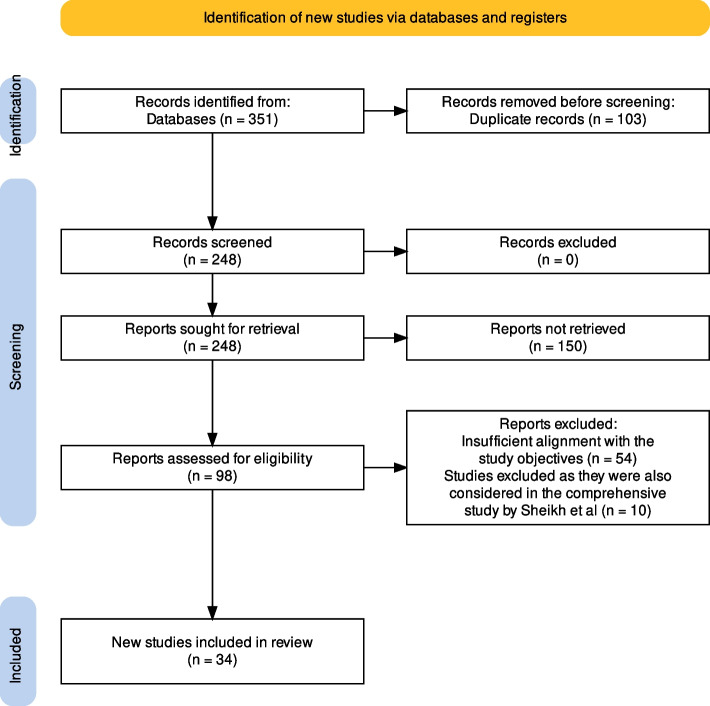
PRISMA 2020 flow diagram

We initiated our systematic review and meta-analysis procedure with the identification of 351 records from two databases: PubMed and Scopus. After this initial identification, we removed 103 duplicate records, leaving us with 248 unique records to be screened. All 248 records were carefully evaluated. However, 150 reports were not retrieved for a detailed evaluation as their titles and abstracts were found to be irrelevant to our research question. The remaining 98 reports were thoroughly assessed for their eligibility. After meticulous review and exclusion of reports that didn’t meet our criteria, we were left with 44 studies. Among these, 10 studies [18–27] were part of the Golestan Cohort. To avoid bias and potential data overlap, we decided to include only one comprehensive study by Sheikh et al. [28] from the Golestan Cohort that encapsulates the data of all 10 studies. A flow diagram detailing this process is included for further clarity (Fig. 1).

### Study characteristics

The studies included in this systematic review and meta-analysis encompass various research designs, cancer types, data collection methods, geographical locations, and periods. These key characteristics are summarized in Table 1. In terms of study design, our review incorporated a mix of case–control studies, cross-sectional studies, and cohort studies. The majority of studies employed a case–control or cross-sectional design, with notable examples being Mahouri (2007), Abdolahinia (2021), Ahmadi (2012), and Akbari (2015) [29–32]. Cohort studies included in our review were Moghadam (2021), Rafati (2019), Samadi (2007), and Sheikh (2020), among others [28, 33–35]. Regarding cancer types, the studies focused on a variety of cancers. Some studies, like Mahouri (2007) and Rafati (2019), focused on breast cancer [29, 34], while others, such as Abdolahinia (2021), Ahmadi (2012), and Akbari (2015), investigated bladder cancer [30–32]. Several studies, like Hadji (2021) and Momtahen (2009), explored multiple cancer types [36, 37]. The provinces where the studies were conducted cover a broad geographical range across Iran, including Hormozgan, Kerman, Mazandaran, Shiraz, Golestan, Tehran, Fars, and others. A notable study by Hadji (2021) and Hosseini (2022) was conducted in 10 provinces [36, 38]. The study periods also varied widely, with some studies conducted as far back as the 2006s (Tabei, 2006) and as recent as 2022 (Hosseini, 2022) [38].

**Table 1:** Characteristics of studies included in systematic review of tobacco use prevalence in Iranian cancer patients

									**Type of consumption**				
**Study**	**Study design**	**Cancer type**	**Data collection method**	**Study province**	**Study period**	**Smokers (total patients)**	**Males (smokers)**	**Females (smokers)**	**Cigarette (N)**	**Naas (N)**	**Waterpipe (N)**	**Pipe (N)**	**Other (N)**
Abdolahinia 2021 [30]	Case–control	Bladder	Interview	Kerman	2020	72 (100)	-	-	72	-	3	-	-
Ahmadi 2012 [31]	Cross-sectional	Bladder	Questionnaire	Mazandaran	2010–2011	52 (108)	-	-	-	-	-	-	-
Akbari 2015 [32]	Case–control	Bladder	Questionnaire	Shiraz	2012–2013	102 (198)	-	-	-	-	-	-	-
Alipour fayez 2020 [39]	Cross-sectional	Lung	Questionnaire	Tehran	2016–2017	15 (31)	-	-	15	-	-	-	-
Aminisani 2016 [40]	Cross-sectional	Crc	Questionnaire	Mazandaran	2007–2013	23 (157)	97 (21)	60 (2)	-	-	-	-	23
Andishe-tadbir 2010 [41]	Cross-sectional	Hnscc	Cancer registry	Fars	2000—2007	66 (148)	-	-	44	-	20	-	2
Azimi 2018 [42]	Case–control	Hnscc	Questionnaire	Tehran	2016–2017	43 (148)	-	-	30	-	-	-	13
Hadji 2021 [36]	Case–control	Multiple^a^	Questionnaire	10 providences	2014–2020	2064 (6598)	-	-	1697	56	300	11	-
Hosseini 2022 [38]	Case–control	Lung	Interview	10 provinces	2016—2020	409 (658)	501 (398)	157 (11)	409	-	76	-	253
Khademi 2012 [43]	Cross-sectional	Upper gi	Questionnaire	Tehran	2002–2009	31 (87)	-	-	31	-	-	-	-
Khoshbaten 2010 [44]	Cross-sectional	Esophageal	Questionnaire	Tehran	-	22 (100)	-	-	-	-	-	-	-
Mafi 2012 [45]	Cross-sectional	Hnscc	Questionnaire	Tehran	1995- 2010	185 (262)	219 (177)	43 (8)	185	-	-	-	-
Mahouri 2007 [46]	Case–control	Breast	Questionnaire	Hormozgan	2000–2002	67 (168)	-	-	-	-	-	-	-
Mashhadi 2011 [47]	Cross-sectional	Esophageal	Questionnaire	Sistan-baluchestan	2005–2010	143 (175)	-	-	-	-	-	-	-
Masjedi 2013 [48]	Case–control	Lung	Questionnaire	Tehran	2002–2005	199 (242)	178 (161)	64 (38)	199	-	-	-	-
Moghadam 2021 [33]	Cohort	Gastric	Questionnaire	Kerman	2001–2016	91 (339)	-	-	91	-	-	-	-
Mohebbi 2020 [49]	Case–control	Hnscc	Questionnaire	10 provinces	2016 -2019	432 (663)	-	-	371	-	61	-	-
Momayez 2021 [50]	Case–control	Pancreatic	Questionnaire	Tehran	2012 -2018	113 (470)	284 (104)	186 (9)	113	-	-	-	-
Momtahen 2009 [37]	Cross-sectional	Multiple^b^	Questionnaire	Tehran	1995- 2005	45 (450)	-	-	-	-	-	-	-
Motovali-bashi 2012 [51]	Cross-sectional	Lung	Interview	Tehran	2009–2011	28 (65)	-	-	-	-	-	-	-
Naghibzadeh 2016 [52]	Case–control	Crc	Interview	Kerman	2014	51 (175)	-	-	51	-	-	-	-
Nikbakht 2015 [53]	Cross-sectional	Crc	Questionnaire	Mazandaran	2007–2012	53 (120)	75 (51)	45 (2)	34	-	19	-	-
Pournaghi 2018 [54]	Case–control	Esophageal	Interview	North khorasan	2013–2015	24 (96)	-	-	-	-	-	-	-
Rafati 2019 [34]	Cohort	Breast	Questionnaire	Kerman	2000–2015	53 (140)	-	-	-	-	-	-	-
Sadjadi 2013 [55]	Cohort	Gastric	Questionnaire	Ardabil	-	62 (129)	-	-	-	-	-	-	-
Saedi 2009 [56]	Cross-sectional	Esophageal	Interview	Tehran	1997–2007	400 (453)	410 (361)	43 (39)	-	-	-	-	-
Salehi 2011 [57]	Cross-sectional	Bladder	Cancer registry	Fars	2007- 2009	109 (216)	-	-	85	-	24	-	-
Samadi 2007 [35]	Cohort	Upper gi	Interview	Ardabil	2000–2004	135 (352)	-	-	-	-	-	-	-
Sheikh 2020 [58]	Cohort	Multiple^c^	Interview	Golestan	2004–2019	472 (1833)	-	-	-	-	-	-	-
Shivappa 2015 [59]	Case–control	Esophageal	Interview	Kurdistan	-	17 (47)	-	-	-	-	-	-	-
Simonian 2018 [60]	Case–control	Crc	Interview	Isfahan	2014 -2015	37 (187)	-	-	-	-	-	-	-
Tabei 2006 [61]	Cross-sectional	Gastric	Interview	Fars	2003	90 (177)	-	-	-	-	-	-	-
Tarrahi 2009 [62]	Cohort	Lung	Interview	Southern iran	2002–2007	160 (238)	-	-	-	-	-	-	-
Vazirinezhad 2020 [63]	Case–control	Gi	Interview	Kerman	2018	25 (95)	-	-	-	-	-	-	-

*Abbreviations*: *CRC* colorectal cancer, *HNSCC* head and neck squamous cell carcinoma, *GI* gastrointestinal

^a^Head and neck, bladder, lung, colon and rectum cancers

^b^Ovarian, uterine corpus or uterine cervix cancers

^c^Laryngeal, bladder, liver, panceriatic, brain, colon, lung, Gastric and esophageal cancers

### Syntheses

**Fig. 2:**
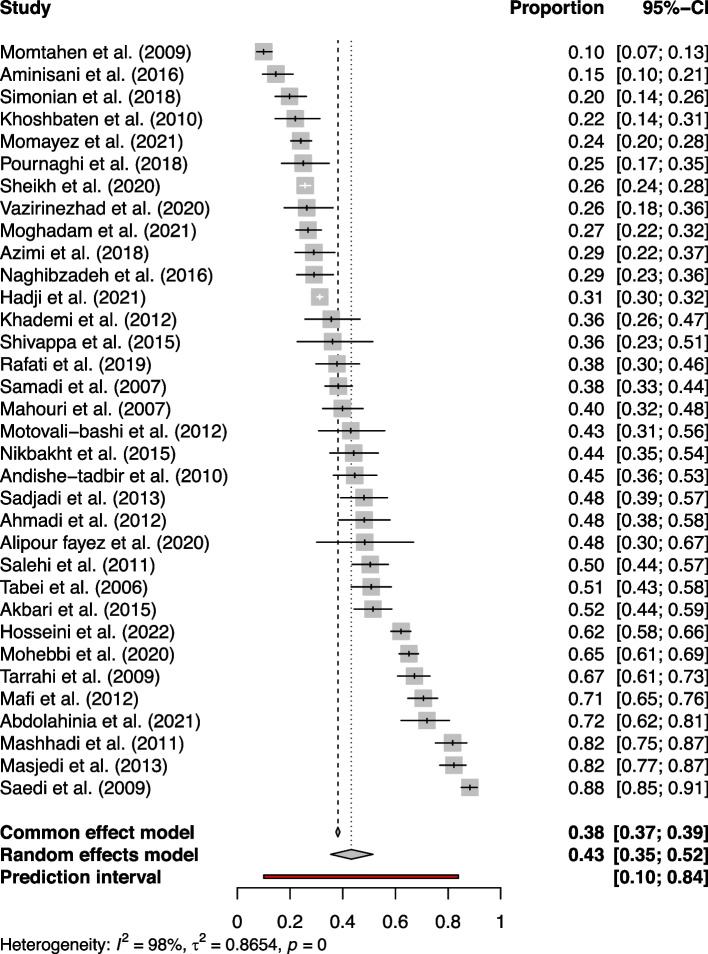
Forest plot of studies included in meta-analysis of tobacco use prevalence in Iranian cancer patients

We included 34 studies with 15,425 patients and 5,890 smokers. The individual tobacco consumption proportions reported ranged from 10.00% (Momtahen et al., 2009) [37] to 82.00% (Masjedi et al., 2013) [48]. Based on study design, 6 cohort, 14 case–control and 14 cross-sectional studies were involved in our analysis. The analysis also considered various cancer types, including 4 bladder, 2 breast, 4 colorectal, 5 esophageal, 3 gastric, 1 gastrointestinal, 4 head and neck squamous cell (HNSC), 5 lung, 1 pancreatic, 2 upper gastrointestinal cancers were included in our analysis. Additionally, 3 studies considered multiple cancer types. The overall proportion of tobacco consumption across all studies, as per the random effects model, was found to be 43.29% (CI95% 35.42%-51.52%, τ^2^ = 0.86, I^2^ = 98.0%) (Fig. 2). The Egger’s test *p*-value was 0.064, suggesting that there was no strong evidence of publication bias, although the intercept value on the funnel plot was 3.57.

### Associations between cancer types and tobacco use

Table 2 presents the outcomes from a comprehensive multiple logistic regression meta-analysis, focusing on the association between cancer type, cigarette smoking, and the prevalence of tobacco use among cancer patients. The analysis highlights a significant heterogeneity (I^2 = 93.792) and reveals distinct associations based on cancer type. The colorectal cancer is significantly associated with lower odds of tobacco use, while laryngeal cancer shows a contrary, positive association. Additionally, the influence of cigarette smoking on its own is significantly affirmed, and its interaction with cancer type elucidates a complex relationship, underscoring the multifaceted dynamics between cancer diagnoses, smoking habits, and their implications for patient health.

**Table 2:** Multiple logistic regression meta-analysis

Model	Term	Estimate	Standard Error	Statistic	*P*	*I*^*2*^	*tau*^*2*^	*H*^*2*^
**Cancer type**						93.792	0.474	16.109
	Intercept	0.150	0.321	0.466	0.645			
	Brain	-1.332	0.804	-1.656	0.110			
	Breast	-0.605	0.595	-1.018	0.319			
	CRC	-1.274	0.454	-2.806	**0.010**			
	Esophageal	-0.265	0.420	-0.630	0.535			
	Gastric	-0.684	0.449	-1.522	0.141			
	Head and neck	-0.220	0.519	-0.424	0.675			
	Laryngeal	0.905	0.846	1.069	0.295			
	Liver	-0.873	0.800	-1.091	0.286			
	Lung	0.259	0.487	0.533	0.599			
	Pancreatic	-1.205	0.599	-2.013	0.055			
**Cigarette**						94.849	0.455	19.415
	Intercept	-0.845	0.374	-2.256	0.051			
	Cigarette	0.009	0.004	2.391	**0.040**			
**Cancer type + Cigarette**						63.553	0.060	2.744
	Intercept	-0.297	0.259	-1.146	0.316			
	Cigarette	0.009	0.002	4.906	**0.008**			
	CRC	-0.663	0.309	-2.146	0.098			
	Gastric	-1.521	0.349	-4.360	**0.012**			
	Head and neck	-0.544	0.274	-1.983	0.118			
	Lung	0.072	0.345	0.208	0.846			
	Pancreatic	-1.864	0.349	-5.349	**0.006**			

### Subgroup analysis

**Fig. 3:**
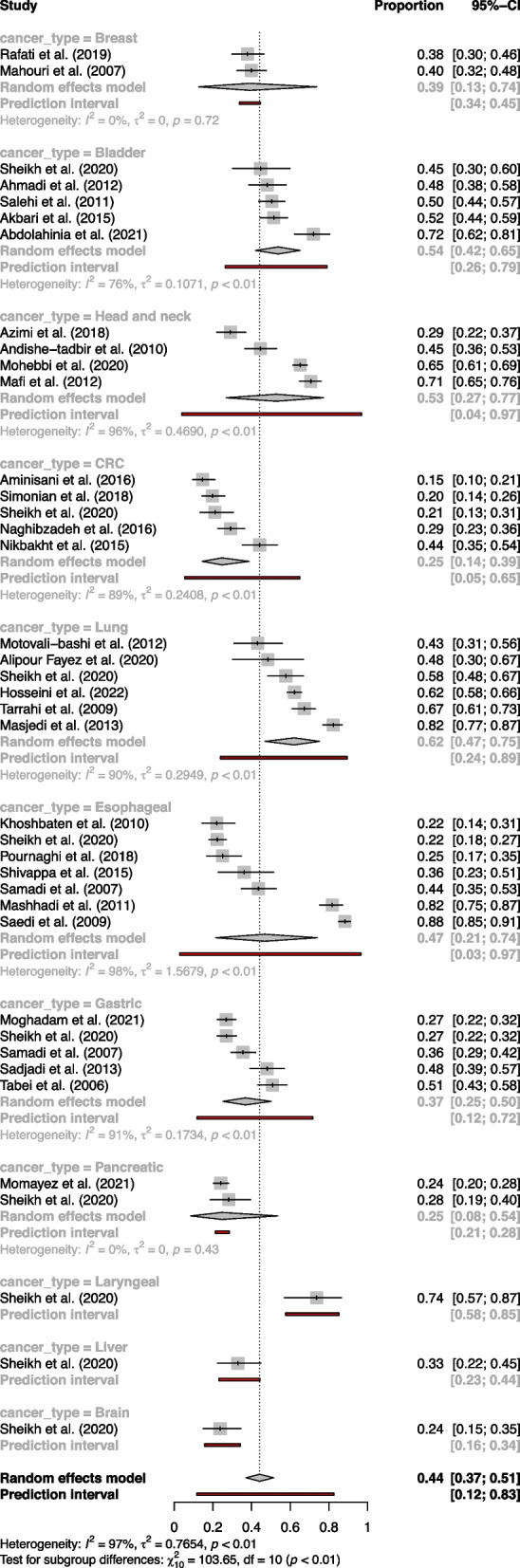
Forest plot of cancer type subgroup analysis in meta-analysis of tobacco use prevalence in Iranian cancer patients

The subgroup analysis, which categorizes studies by the type of cancer, showed varying percentages of tobacco consumption for each cancer type. The highest percentage of tobacco use was observed in the laryngeal cancer subgroup at 73.68% (57.63%-85.22%), albeit based on only one study. This was followed by lung cancer at 62.05% (47.00%-75.09%), bladder cancer at 53.74% (41.99%-65.08%) and head and neck cancer at 52.73% (26.80%-77.27%). Tobacco consumption was lower in esophageal cancer at 46.77% (21.35%-73.98%), breast cancer subgroup at 38.96% (12.64%-73.80%), gastric cancer at 36.80% (25.20%-50.16%), and CRC at 24.63% (14.46%-38.72%). The heterogeneity was also found to differ between these cancer type subgroups (τ^2^ = 0.7654; τ = 0.8749; I^2^ = 96.8%) (Fig. 3).

**Fig. 4:**
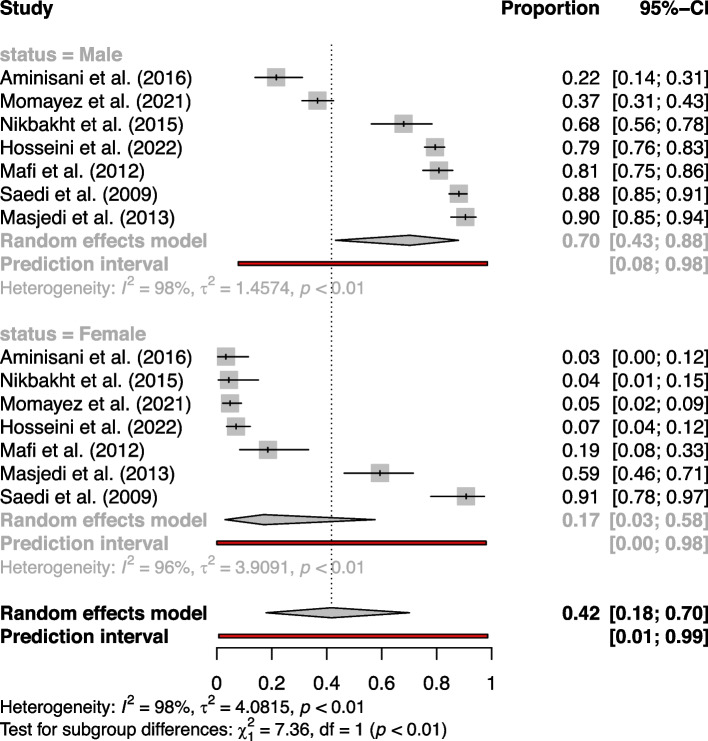
Forest plot of gender subgroup analysis in meta-analysis of tobacco use prevalence in Iranian cancer patients

On the other hand, the analysis based on gender, encompassing a total of 2362 patients and 1382 smokers, revealed a significant difference in tobacco consumption rates. Overall estimated tobacco consumption was 70.22% (95%CI: 43.21%-87.96%) in men and 17.11% (95%CI: 3.04%-57.59%) (Fig. 4). The study by Saedi et al. (2009) [56] reported the highest rate of tobacco consumption at 90.70% (77.86%-97.41%) in women with esophageal cancer, while the study by Masjedi et al. (2013) [48] reported the highest tobacco consumption at 90.45% (85.15%-94.34%) in men with lung cancer. Conversely, the lowest for both sexes was reported by Aminisani et al. (2016) [40], standing at 21.65% (13.93%-31.17%) and 3.33% (0.41%-11.53%), in men and women with CRC, respectively.

**Fig. 5:**
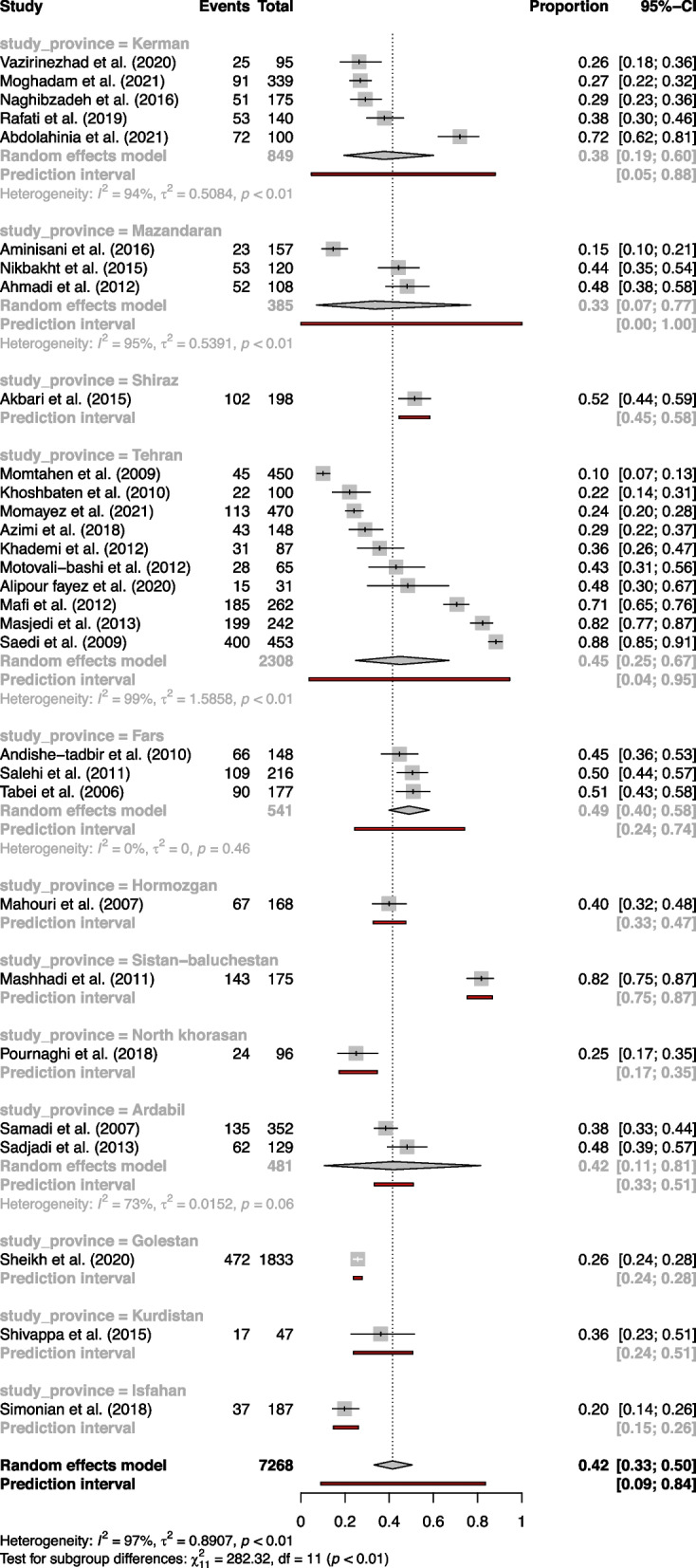
Forest plot of province subgroup analysis in meta-analysis of tobacco use prevalence in Iranian cancer patients

In terms of geographical location, varying tobacco consumption rates were observed across different provinces of Iran. The studies reported a wide range of tobacco consumption proportions, from 10.00% (Momtahen et al., 2009) [37] to 88.30% Saedi et al. (2009) [56]. When the studies were collectively analyzed using a random effects model, the estimated overall rate of tobacco consumption was 41.50% (33.12%-50.41%). A subgroup analysis was conducted based on the province where each study was conducted. It showed a considerable variation in the rate of tobacco consumption across the provinces. For instance, the highest proportion of tobacco consumption was found in Sistan-baluchestan at 81.71%, while the lowest was recorded in North Khorasan at 25.00% (Fig. 5).

**Fig. 6:**
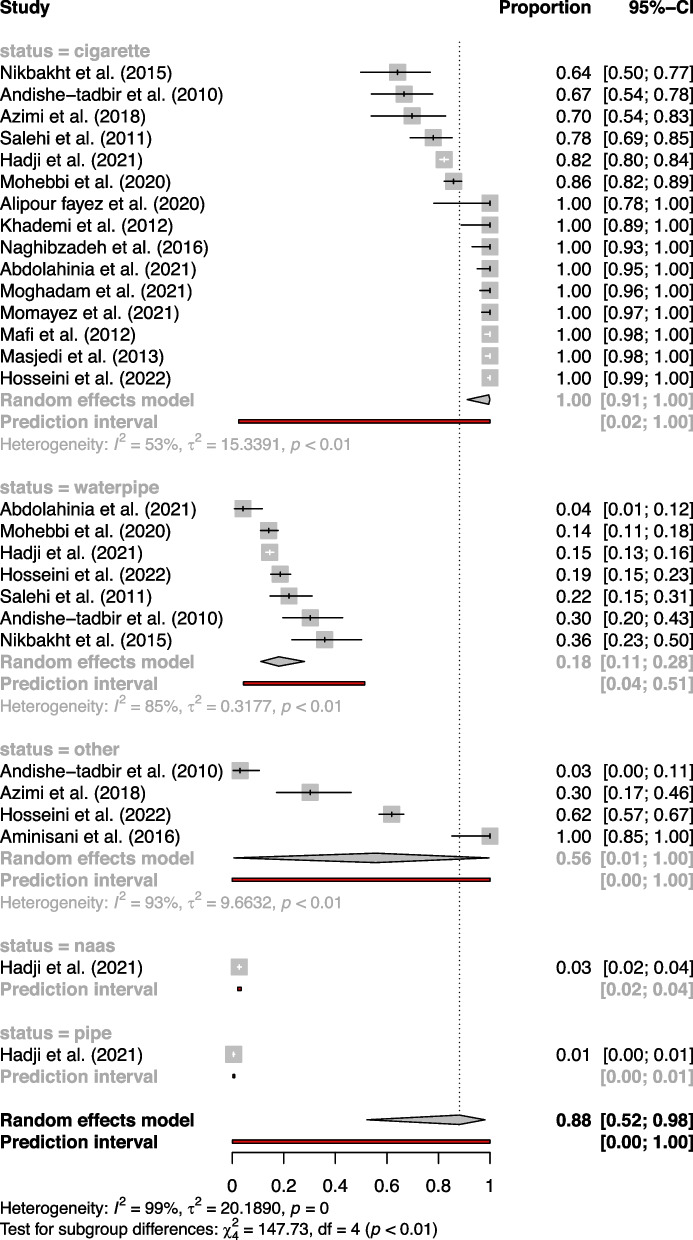
Forest plot of tobacco type subgroup analysis in meta-analysis of tobacco use prevalence in Iranian cancer patients

When divided based on tobacco consumption habits, the subgroups showed differences. The prevalence of cigarette smoking was notably high, with a proportion of 99.51% (91.16%—99.97%) across 15 studies. For waterpipe smoking, the prevalence was 18.09% (11.14%—28.01%) across seven studies, and other forms of tobacco use had a prevalence of 55.67% (0.65%—99.59%) across four studies. Usage of 'Naas' and 'Pipe' were evaluated in single studies and showed much lower prevalence rates, 2.71% (2.09%—3.51%) and 0.53% (0.03%—0.96%) respectively (Fig. 6).

### Results of sensitivity analysis

When potential outliers identified through both dmetar and GOSH methods were removed, the overall proportion did not change considerably, remaining at 0.41. However, the 95% confidence interval narrowed to [0.37–0.45] and [0.35–0.47] respectively, and the heterogeneity decreased notably, with I^2 values of 0.71 and 0.96 respectively. This suggests the overall result was robust but the heterogeneity was driven by a few outlying studies (Table 3).

**Table 3:** The sensitivity analysis after removed outlier studies

Analysis	*Proportion*	95%CI	95%PI	*I*^*2*^	95%CI
Main Analysis	0.43	0.35–0.52	0.1–0.84	0.98	0.98–0.98
Infl. Cases Removed with dmetar^a^	0.41	0.37–0.46	0.27–0.57	0.73	0.57–0.83
Infl. Cases Removed with GOSH^b^	0.43	0.35–0.51	0.12–0.81	0.97	0.96–0.97

^a^Removed as outliers: Abdolahinia et al. (2021) [30], Aminisani et al. (2016) [40], Hadji et al. (2021) [36], Hosseini et al. (2022) [38], Khoshbaten et al. (2010) [44], Mafi et al. (2012) [45], Mashhadi et al. (2011) [47], Masjedi et al. (2013) [48], Moghadam et al. (2021) [33], Mohebbi et al. (2020) [49], Momayez et al. (2021) [50], Momtahen et al. (2009) [37], Pournaghi et al. (2018) [54], Saedi et al. (2009) [56], Sheikh et al. (2020) [58], Simonian et al. (2018) [60], Tarrahi et al. (2009) [62]

^b^Removed as outliers: Hadji et al. (2021) [36], Mashhadi et al. (2011) [47], Masjedi et al. (2013) [48], Momtahen et al. (2009) [37], Motovali-bashi et al. (2012) [51], Sheikh et al. (2020) [62]

## Discussion

Our comprehensive systematic review and meta-analysis has shed light on the intriguing landscape of tobacco consumption in relation to various cancer types in Iran. The overall tobacco consumption proportions, calculated using a random effects model across all studies, settled at 42%. This means that almost half of the participants in these studies reported consuming tobacco. However, our statistical investigation also brought to light significant heterogeneity within the studies, pointing towards considerable variations in the tobacco consumption patterns.

Looking at the subgroup analysis, it's evident that tobacco consumption varies significantly across different cancer types and demographic groups. For instance, patients with laryngeal cancer showed the highest proportion of tobacco consumption (73%), while colorectal cancer patients showed the lowest (24%). When we examined tobacco use based on gender, we found males to exhibit a higher rate of consumption. Geographical disparities also emerged, with Sistan-baluchestan province reporting the highest rate of tobacco consumption and Tehran the lowest.

In terms of tobacco consumption habits, cigarette smoking emerged as the most prevalent, featuring in 15 studies with a proportion of 99%. Other forms of tobacco use, including waterpipe smoking and the use of ‘Naas’ and ‘Pipe’, showed varying levels of prevalence.

The high prevalence of ever smokers among cancer patients is notable, emphasizing the extensive impact tobacco has had on this population. Recognizing ever smokers—individuals who have smoked at any point in their lifetime—allows for a comprehensive understanding of tobacco exposure and its potential role in cancer progression and outcomes. This understanding is crucial for developing effective public health strategies, designing tailored interventions for tobacco cessation, and informing clinical guidelines aimed at reducing tobacco-related risks among cancer patients.

In 2021, the STEPwise approach to chronic disease risk factor surveillance (STEPS) survey provided comprehensive insights into the prevalence of tobacco use among Iranian adults, revealing distinct variations by gender, age, and usage patterns [64]. Overall, the prevalence of current tobacco smoking among the Iranian adult population was reported at 14.01% (13.56–14.48). When disaggregated by gender, a stark contrast emerges, with 25.88% (25.03–26.75) of men and only 4.44% (4.09–4.82) of women reported as current tobacco users. This gender disparity extends to specific tobacco products, with 19.95% (19.17–20.75) of men and 0.77% (0.62–0.95) of women identified as current cigarette smokers, and 5.56% (5.12–6.03) of men compared to 3.64% (3.33–3.98) of women reported as current hookah smokers.

The survey further delves into the age-related patterns of tobacco use, highlighting that cigarette smoking among men peaks at 26.43% (24.47–28.48) in the 45–54 year age group before declining, while the use of hookah shows its highest prevalence among both men (11.03% [9.66, 12.56]) and women (5.87% [4.98, 6.9]) aged 25–34. This age-specific data suggests a pronounced variation in smoking habits across different life stages. Additionally, the prevalence of second-hand smoking exposure at home was significantly high, with 24.64% (24.05–25.24) overall prevalence, showing higher exposure rates among women (27.38% [26.59–28.18]) compared to men (20.26% [19.39–21.17]). The geographical analysis of tobacco prevalence across the 31 provinces of Iran unveiled significant variations, further emphasizing the need for targeted public health interventions. The survey's findings underscore the persistently high rates of tobacco consumption in Iran, reflecting the pressing need for enhanced tobacco control policies and interventions that are sensitive to gender, age, and regional disparities. Given the elevated rate of tobacco use among patients with cancer at approximately 42% [36–42, 48, 56, 64–66], as identified in our study, these data call for urgent public health actions to address tobacco use as a critical risk factor for cancer and other non-communicable diseases in Iran.

To make sense of the different risks associated with various types of cancer in connection with tobacco use, a previous meta-analysis systematically examined the relative risks. The findings indicated that the highest risks were found in lung, laryngeal, and pharyngeal cancers, with upper digestive tract and oral cancers following closely behind [65]. In our more detailed subgroup analysis, we examined the studies based on cancer type, unveiling differing levels of tobacco consumption for each. We found that tobacco use was most prevalent in patients with laryngeal cancer. This group was closely followed by those with lung, head and neck, and bladder cancers. On the other hand, we noticed lower tobacco use in patients with breast, esophageal, gastric, and colorectal cancers.

Tobacco consumption in Iran presents a complex pattern of regional disparities, with distinct differences observed between the general population and cancer patients. As per the STEPwise report, in 2011, the north-western provinces, including West-Azerbaijan, East-Azerbaijan, Ardabil, Kordestan, Zanjan, Qazvin, and Gilan, recorded the highest rates of current tobacco smoking. However, by 2016, the epicenter of highest prevalence had migrated to Hamadan and Qazvin. Throughout these years, the western provinces, especially the north-west, consistently reported higher tobacco use compared to their eastern counterparts. In 2016, the southern provinces of Bushehr, Fars, and Hormozgan, along with Sistan and Baluchestan in the south-east and Razavi-Khorasan in the north-east, also emerged with high prevalence rates [66].

In examining cancer patients, the pattern of tobacco consumption across different regions reveals nuanced insights. Tehran is notable for its range of tobacco use among cancer patients, reported between 10% and 82.23%. This upper figure aligns with expectations when considering the elevated risk smoking poses for lung cancer, especially given the 26% smoking prevalence among Iranian men. Kerman also exhibits significant tobacco use among cancer patients, with rates from 26.32% to 72%. Mazandaran presents a varied scenario, where tobacco use among cancer patients ranges from 14.65% to 48.15%. Notably, the highest observed prevalence rates among cancer patients are in Sistan-Baluchestan (81.71%) and Tehran (82.23%), underscoring the link between tobacco exposure and cancer incidence in these regions. Therefore, while the general population in north-western provinces and some southern areas shows elevated tobacco consumption, Tehran and Sistan-Baluchestan demonstrate the most pronounced prevalence among cancer patients, reflecting the known risk factors associated with smoking.

We recognize that regional variations in tobacco use among cancer patients may be influenced by the types of cancers predominantly studied within those regions. Given the strong association between smoking and certain types of cancer, such as lung and larynx cancers, the prevalence of smoking is likely higher in regions where these cancers are more frequently studied. This potential confounding factor suggests that our observed regional differences in smoking prevalence might not solely reflect geographical variations in smoking behavior, but also the specific cancer types investigated in each region.

Tobacco consumption in Iran, both in the general populace and among cancer patients, exhibits a distinct pattern when dissected by the type of tobacco product used. In the general population, hookah use is relatively prevalent, with 3.6% of women and 5.6% of men engaging in this practice. Sistan and Baluchistan stand out with the highest usage of smokeless tobacco. The prevalence of men who have ever smoked cigarettes varies widely, from a low of 13.28% in South Khorasan to a high of 39.02% in Qazvin. Similarly, the prevalence of men who have ever used hookah ranges from 3.68% in Kermanshah to 22.38% in Isfahan. Among women, the prevalence of ever smoking cigarettes is generally low, peaking at 1.59% in Tehran, while the current use of hookah ranges from zero in Ardabil and West Azerbaijan to a significant 15.27% in Sistan and Baluchistan. Pipe smoking and smokeless tobacco, however, find little favor among the Iranian population [64].

In contrast, among cancer patients, the landscape of tobacco consumption shifts noticeably. Cigarette smoking emerges as the dominant form, with prevalence rates spanning from 64.15% to a full 100% in various studies. Waterpipe or hookah smoking, while less prevalent than cigarette smoking, still shows a considerable range of 4.17% to 35.85%. Other forms of tobacco use, including smokeless tobacco and pipe smoking, are relatively rare, with prevalence rates of 3.03% to 30.23% and 0.53% respectively. In essence, while cigarette smoking is the most common form of tobacco use across both the general population and cancer patients, hookah use is also a significant concern, particularly in certain provinces. Other forms of tobacco use, such as smokeless tobacco and pipe smoking, are less prevalent.

### Strengths and limitations

Our findings, enriched by detailed subgroup analyses across cancer types, gender, geographic regions, and tobacco use modalities, underscore the critical public health implications of tobacco use among cancer patients, revealing a prevalence markedly higher than in the general population. Our study's limitations extend to include a restricted research scope as we relied solely on two databases, and due to a lack of direct studies, we had to use data embedded within these studies. The sustained high heterogeneity detected throughout our analyses suggests that numerous unexplored factors, beyond the boundaries of our study, could have an influential role. These factors might encompass socio-economic conditions in different provinces, distinct cultural practices, the effectiveness of cancer control programs, and the accuracy and accessibility of cancer registries. Additionally, the observed heterogeneity might be attributed to discrepancies in the types of studies we sourced, the specific cancer types analyzed, and their association with smoking. However, it is noteworthy to mention that because our meta-analysis was focused on evaluating prevalence, the high heterogeneity could be deemed acceptable due to these aforementioned reasons. Such a degree of heterogeneity may not present as a critical issue like when we calculate Odds Ratios (OR) or Relative Risks (RR). An important limitation of our study is that the observed regional variations in smoking prevalence among cancer patients could be influenced by the selection of cancer types studied in each region. This aspect might have introduced a bias towards higher smoking prevalence in regions focusing on cancers strongly associated with smoking. Future studies should aim to disentangle the effects of regional cancer type distribution from true variations in smoking behavior.

## Conclusion

In conclusion, our systematic review and meta-analysis provide valuable insights into the link between tobacco consumption and various cancer types in Iran, revealing considerable heterogeneity in consumption patterns across different demographics, geographical regions, and cancer types. Notably, the rate of tobacco consumption among cancer patients is threefold higher than in the general Iranian population. The study also unveils a concerning picture of the prevalent use of cigarettes and the variable use of other forms of tobacco, including waterpipe smoking, ‘Naas’, and ‘Pipe’, among cancer patients.

## Data Availability

Data will be available upon reasonable request from the corresponding author.
